# A registry study of nursing assessments, interventions and evaluations according to nutrition for persons living in municipal residential care homes

**DOI:** 10.1002/nop2.144

**Published:** 2018-04-16

**Authors:** Anja Backlund, Olga Holmbeck, Christine Kumlien, Malin Axelsson

**Affiliations:** ^1^ Faculty of Health and Society Department of Caring Science Malmö University Malmö Sweden; ^2^ Department of Cardio‐Thoracic and Vascular Surgery Skane University hospital Malmö Sweden

**Keywords:** intervention, nurses, nursing, nutrition, Sweden

## Abstract

**Aim:**

The aim was to explore planned nursing interventions and evaluations of such interventions, in older people at risk for malnutrition living in municipal residential care homes.

**Designs:**

A registry study.

**Methods:**

The study was conducted using data from the Swedish national quality registry Senior Alert. Data on all persons assessed and registered in Senior Alert living in municipal residential care homes in a mid‐sized town between January and December 2014 were subjected to statistical analysis.

**Results:**

In total, 677 nutritional risk assessments were performed among the participants (*N* = 587), who were between 65‐109 years. A larger proportion of women were estimated as being at risk for malnutrition compared with men. The three most common prescribed nursing interventions were nutritional treatment, dietary support and weight control; however, interventions were not prescribed for all participants at risk for malnutrition. Lesser than 50% of the interventions were evaluated, with dietary support, pharmaceutical review and weight control the three most likely to be evaluated. Further, planned interventions for participants at risk of malnutrition were implemented more often for men than for women.

## 1 INTRODUCTION

Malnutrition among older people living in municipal residential care homes is a common problem (Borgström Bolmsjö, Jakobsson, Mölstad, Östgren, & Midlöv, 2015; Carlsson, Gustafson, Eriksson, & Håglin, 2009; Meijers, Halfens, van Bokhorst‐de van der Schueren, Dassen, & Schols, 2009). Undetected and untreated malnutrition means a deterioration of quality of life, prolonged recovery and an increased risk for morbidity and mortality. Therefore, an early detection of older people at risk of malnutrition is essential to prevent these risks (Norman, Pichard, Lochs, & Pirlich, 2008) and to promote quality of life (Volkert, 2013). Malnutrition in this group also has an impact on a societal level, for example, in terms of increased healthcare costs (Lorefält, Andersson, Wirehn, & Wilhelmsson, 2011). Therefore, the current study focused on nursing interventions and evaluations for older people at risk of malnutrition living in municipal residential care homes.

## 2 BACKGROUND

The ageing population is increasing worldwide (Rechel, Doyle, Grundy, & McKee, 2009). Estimates indicate that the proportion of the population older than 60 years will double between the years 2000 and 2050, rising from 11% to 22% and representing an expected increase from 605 million to 2 billion (World Health Organization, 2014). An ageing population is a socio‐economic challenge (World Health Organization, 2003), that requires appropriate health and social policies (Rechel et al., 2009). Among the factors that are important to consider is the probability that malnutrition will increase among persons living in municipal residential care homes. Importantly, recognizing and treating malnutrition in older people can be difficult; therefore, greater awareness of this problem is needed (Meijers et al., 2009).

The prevalence of malnutrition among older people living in municipal residential care homes varies across countries (Blössner & Onis, 2005; Caselato‐Sousa, Guariento, Crosta, da Silva Pinto, & Sgarbieri, 2011; Leslie et al., 2013) and the estimates vary between 17% and 30% (Borgström Bolmsjö et al., 2015; Carlsson et al., 2009; Verbrugghe et al., 2013; Volkert, Saeglitz, Gueldenzoph, Sieber, & Stehle, 2010). Regarding prevalence of risk for malnutrition, the estimations fluctuate between 28% and 59% (Borgström Bolmsjö et al., 2015; Carlsson et al., 2009; Verbrugghe et al., 2013). However, it has been argued that there is a lack of international consensus on diagnostic criteria for malnutrition, which may explain why only 150–300 inpatient cases per year were diagnosed with malnutrition, according to International Statistical Classification of Diseases (ICD) 10 in Sweden over the period 1998–2012 (Swedish Council on Technology Assessment, 2014).

The World Health Organization (WHO) defines malnutrition as the extent to which a person's body weight deviates from the mean weight in a reference population (Blössner & Onis, 2005). Malnutrition can also be evaluated using various assessment instruments, such as the Mini Nutritional Assessment Short Form (MNA‐SF) (Kaiser et al., 2009), Malnutrition Universal Screening Tool (MUST) (Scott, 2008), Mini Nutritional Assessment (MNA) (Guigoz, Vellas, & Garry, 1996) and Nutritional Risk Screening (NRS‐2002) (Kondrup, Allison, Elia, Vellas, & Plauth, 2003). MNA has been found to offer a higher degree of accuracy than MUST and NRS‐2002 for detection of malnutrition in older people (Holst et al., 2013).

Malnutrition in older people can be related to old age (Saletti et al., 2005), low physical activity and decreased cognitive ability, as well as insufficient protein intake or poor chewing and swallowing function (Blaum, Fries, & Fiatarone, 1995). Depression and reduced quality of life affect the appetite and can lead to an increased tendency to develop malnutrition; moreover, dementia and various states of confusion can magnify the impact on weight loss and malnutrition. Reduced handgrip strength, oral fungal infection, a low albumin level, high CRP and decreased cognitive function are all associated with risk of mortality within 12 months; and all can appear in connection with malnutrition (Holst et al., 2013). Moreover, an institutional environment is an independent risk factor for malnutrition in older people living in municipal residential care homes (Strathmann et al., 2013). Importantly, only a fraction of those in need of nutritional therapy actually receive such treatment due to the lack of recognition of the early signs of risk of malnutrition, as mentioned above. The signs can also appear as muscle dysfunction and low Body Mass Index (BMI) (Norman et al., 2008).

Malnutrition constitutes a significant health risk for older people in terms of increased risk of the following: mortality, morbidity (Johansson, Bachrach‐Lindstrom, Carstensen, & Ek, 2009; Norman et al., 2008; Söderstrom, Rosenblad, Adolfsson, Saletti, & Bergkvist, 2014; reduced quality of life (Luger et al., 2016), suffering due to poor healing of wounds, pressure ulcers, infections and extended convalescence (Norman et al., 2008). The association between the risk of malnutrition and impaired functionality clearly indicates the need for increased awareness of the consequences of malnutrition. Preventing malnutrition and taking early measures to avoid further nutritional and functional deterioration are essential aspects (Stange, Poeschl, Stehle, Sieber, & Volkert, 2013) that can spare people from unnecessary suffering and also reduce the economic costs to society (Guest et al., 2011; Lorefält et al., 2011). This implies that preventive measures and health preservation should be prioritized from both an economic and a social perspective (Rechel et al., 2009). Maintaining a proper nutritional status is important for quality of life among persons living in municipal residential care homes (Norman et al., 2008).

In the current study, the term interventions refers to treatment/therapies, procedures and/or actions within the scope of nursing that are given to or performed on/with an individual in a specific situation to improve the person's condition and achieve a better health outcome (Sidani & Braden, 2011;. Nurses have a central role in identifying and taking responsibility for people at risk of malnutrition and for ensuring that nursing interventions are introduced at an early stage (Holmen, Robertsson, & Wijk, 2006).

For nurses working to prevent malnutrition, it is imperative to recognize its early signs (Holmen et al., 2006; Kaiser et al., 2009), to understand the associated risk factors and to know how to avert them (Lorefält et al., 2011; Norman et al., 2008; Söderstrom et al., 2013). Of further significance is to consider factors influencing older people′ willingness to eat, as this may have an impact on the nutritional status (Wikby & Fagerskiöld, 2004). Improved knowledge among nurses can help prevent malnutrition and slow the progression of weight loss (Lorefält et al., 2011). According to the World Health Organisation, there is some evidence that nutritional supplements can reduce mortality (Rechel et al., 2009); there are also findings that suggest that food enrichment can retard chronic weight loss in people living in municipal residential care homes (Leslie et al., 2013). As nutritional assessments are crucial to detect malnutrition in time (Norman et al., 2008), it is, therefore, vital to both create and use guidelines (Persenius, Hall‐Lord, Bååth, & Wilde Larsson, 2008) and frameworks for early detection in older people (Volkert et al., 2010). For example, the guidelines and recommendations of the European Society for Clinical Nutrition and Metabolism (ESPEN) (Bozzetti & Forbes, 2009) could be applied routinely in a structured manner. Moreover, it can be beneficial to review current evidence‐based care in the field (Bozzetti & Forbes, 2009; Volkert et al., 2010). Another mechanism that can help nurses to work in a structured way to both prevent and evaluate malnutrition and thereby to improve care quality, is the national quality registry tool Senior Alert (Edvinsson, Rahm, Trinks, & Höglund, 2015). Developed in Sweden, Senior Alert is a unique and modern national quality registry (Senior Alert, 2016a) that focuses on population health among older people. The aim of the registry is to enable better health outcomes and reduced costs for society. With regard to nutrition, data from Senior Alert could be used to recognize malnutrition, to initiate interventions and to evaluate interventions (Edvinsson et al., 2015).

Malnutrition is a common health problem in older people leading to increased risk for morbidity and mortality and decreased quality of life. Early detection of malnutrition, planning and evaluation of adequate nursing intervention are crucial in older people to reduce associated risks. However, there is a need for further knowledge about planned nursing interventions used for older people at risk for malnutrition living in municipal residential care homes.

## 3 AIM AND RESEARCH QUESTIONS

The aim of this study was to explore planned nursing interventions and evaluations of such interventions, in older people at risk for malnutrition living in municipal residential care homes:

- What nursing interventions are planned among older people?
- To what extent are planned interventions evaluated?

### 3.1 Design

#### 3.1.1 Study design and setting

A registry study was conducted (Polit & Beck, 2013) based on the national quality registry Senior Alert, which is one of several national quality registries in Sweden. Senior Alert is a tool for quality improvement and safety for older people, focusing primarily on malnutrition, poor oral health, falls, bladder dysfunctions and pressure ulcers. Healthcare professionals working at county hospitals and municipal care settings enter data in Senior Alert after having informed the patient. Each care setting has the legal obligation to inform each person about the data recording in the registry. If a person does not want to participate, he/she can withdraw from the registry (Edvinsson et al., 2015).

### 3.2 Method

#### 3.2.1 Study participants

Included in the study were older people (both men and women) living in 14 municipal residential care homes, where approximately 800 apartments are available, in a mid‐sized town in southern Sweden. Residents of private care homes were excluded.

#### 3.2.2 Data collection

The data collected covered the period January to December 2014. Anonymous data on the following variables were collected: sex, age, BMI, MNA‐score and planned interventions and date for evaluation of planned interventions. Medical history and general condition of the participants are not registered and, therefore, unavailable in the registry.

#### 3.2.3 Assessment instruments

MNA‐SF is a short form of the MNA instrument (Guigoz et al., 1996; Kaiser et al., 2009) and it is used to screen for the risk of malnutrition among the persons registered in Senior Alert (2016b). The MNA‐SF scale also offers advantages for assessment of disabled people with the option of using calf circumference (CC) when BMI cannot be calculated (Kaiser et al., 2009). Items A–F of the MNA‐SF were used to assess nutritional status: A = mode of feeding (0–2 points), B = weight loss (0–3 points), C = mobility (0–2 points), D = stress/acute disease (0–2 points), E = dementia/depression (0–2 points) and F = BMI (0–3 points). Item F can be recorded as either BMI or CC, depending on the feasibility of measurements on a person living in municipal residential care home. By summarizing the MNA‐SF score, nutritional status can be divided into three categories (Guigoz et al., 1996; Kaiser et al., 2009): well nourished, 12–14 points; risk for malnutrition, 8–11 points; and malnutrition, 0–7 points (Guigoz et al., 1996).

#### 3.2.4 Definitions

In the present study, MNA‐SF scores were divided into two groups: <12 indicated at risk of malnutrition, ≥12.0 represented not at risk of malnutrition.

To facilitate analysis of the data, interventions were categorized according to the instructions for MNA‐SF provided by Senior Alert (2016b):

- Dietary support: adaptation of environment for individual meal situation, encouragement and prompting, feeding, planning aids, eating and drinking practice, other intervention.
- Nutritional treatment: snacks, enrichment of food, protein and energy enrichment, texture adaptation, nutritional supplements, adaptation of food according to culture or religion, overnight fasting decreased to a maximum of 11 hr, enteral nutrition, other intervention.
- Registration of nutritional and fluid intake: for <3 days, for >3 days, other intervention.
- Weight control: once a week, at least once every 3 months.
- Oral care: training oral care, assisting with oral care, other intervention ‐oral care.
- Information/Education regarding nutrition.
- Other intervention: malnutrition.
- Care in the final stages of life: malnutrition.
- Declining all preventive interventions: malnutrition (44).

BMI was calculated as weight in kilograms divided by height in metres squared (kg/m²), while the BMI values for the risk assessments were categorized as follows: <22 risk of malnutrition; 22–29, normal weight; >29, overweight.

#### 3.2.5 Statistical analysis

IBM SPSS Statistics for Windows Version 22.0 was used to perform statistical analyses. The descriptive statistics applied were mean, median, percentage and frequencies; and scales used were nominal. Student's *t* test was used to compare subgroups and the Chi‐Square test (χ^2^ test) was used to compare proportions. Logistic regression was used to identify predictors of evaluated interventions. A *p*‐value of ≤.05 was regarded as statistically significant (Marston, 2010; Pallant, 2013).

#### 3.2.6 Ethical consideration

The manager of the municipal residential care homes received both oral and written information about the study. Written consent to use depersonalized data from Senior Alert was obtained from the operational manager of the care homes. The study was approved by the Ethical Review Board of Malmö University (reference number: HS60‐2014/1244:5) and it complied with the Declaration of Helsinki (World Medical Association 2013).

## 4 RESULTS

The results cover the year 2014 and a total of 677 risk assessments of 587 individuals were analysed. Most participants were evaluated once but five participants were evaluated three times and 85 persons were evaluated twice. The age span of the participants was 65–109 years, with a mean age of 86.2 years, women being significantly older than men. A larger proportion of women were at risk of malnutrition compared with men, but the mean score of MNA‐SF did not differ between the sexes (Table 1).

**Table 1:** Description of the study participants

Variables	All *N* = 587	Men *N* = 176	Women *N* = 411	*p*‐values. Comparisons of men and women
Age mean (*SD*)	86.2 (7.3)	83.46 (7.3)	87.36 (6.9)	.001^a^
MNA‐SF mean (*SD*)	10.6 (2.4)	10.85 (2.3)	10.46 (2.4)	.065^a^
Risk for malnutrition *N*(%):				.044^b^
Yes	347 (59.1)	93 (52.8)	254 (61.8)	
No	240 (40.9)	83 (47.2)	157 (38.2)	
BMI mean (*SD*)	24.49 (4.9)	25.56 (4.8)	24.03 (4.8)	.001^a^
BMI groups *N*(%):				.001^b^
22–29 normal	299 (51.0)	103 (58.5)	196 (47.7)	
<22 under	186 (31.7)	36 (20.5)	150 (36.5)	
>29 over	101 (17.2)	37 (21.0)	64 (15.6)	

*SD*, standard deviation; MNA‐SF, Mini Nutritional Assessment Short Form; BMI, Body Mass Index.

a *t* test.

b Chi‐Square test.

### 4.1 Assessments of nutritional status in relation to planned and evaluated interventions

**Figure 1:**
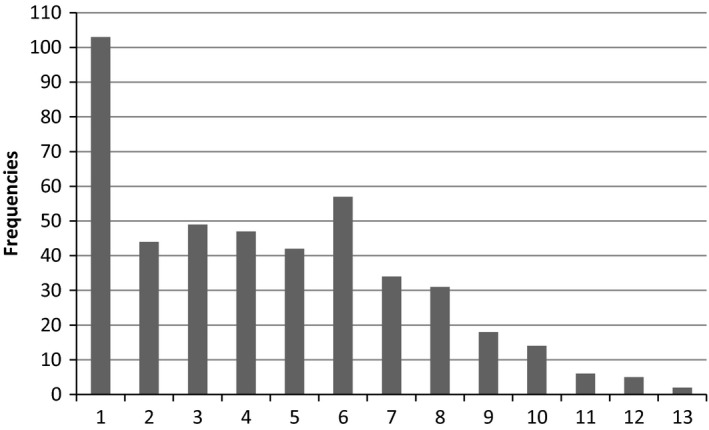
In total, 2018 interventions were planned meaning that some of the risk assessments yielded more than one intervention. Most common was one prescribed intervention per risk assessment

As presented in Table 2, a total of 66.8% of the 677 assessments of nutritional status had planned interventions, of which 27.5% were evaluated. In total 2,018 interventions were planned, meaning that for some participants more than one intervention was planned. For most participants (*N* = 103), one intervention was planned (Figure 1). Table 2 shows that no differences between women and men regarding planned and evaluated interventions were identified. Of the 395 assessments indicating risk for malnutrition, 17.0% did not have any planned interventions. Moreover, 44.0% of the assessments indicating no risk for malnutrition had planned interventions. The proportions of evaluated interventions were 35.7% in participants with risk for malnutrition and 16% among participants without risk for malnutrition. Interventions were more likely to be planned among men at risk of malnutrition compared with women in the same category.

**Table 2:** Nutritional assessments based on MNA‐SF (*N* = 677) in relation to planned and evaluated nursing interventions

Variables	Total *N* = 677	In men *N* = 193	In women *N* = 484	*p*‐values^a^ comparisons between men and women	In persons with risk for malnutrition *N* = 395	In persons without risk for malnutrition *N* = 282	*p*‐values^a^ comparisons between persons with and without risk	In men with risk for malnutrition	In women with risk for malnutrition	*p*‐values^a^ comparisons between persons with and without risk
	*N*(%)	*N*(%)	*N*(%)		*N*(%)	*N*(%)		*N*(%)	*N*(%)	
Planned				.471			.001			.005
Yes	452 (66.8)	133 (68.9)	319 (65.9)		328 (83.0)	124 (44.0)		92 (92.0)	236 (80.0)	
No	225 (33.2)	60 (31.1)	165 (34.1)		67 (17.0)	158 (56.0)		8 (8.0)	59 (20.0)	
Evaluated interventions:				.129			.001			.548
Yes	186 (27.5)	45 (23.3)	141 (29.1)		141 (35.7)	45 (16.0)		33 (33.0)	108 (36.6)	
No	491 (72.5)	148 (76.7)	343 (70.9)		254 (64.3)	237 (84.0)		67 (67.0)	187 (63.4)	

MNA‐SF, Mini Nutritional Assessment Short Form.

a Chi‐Square test.

**Figure 2:**
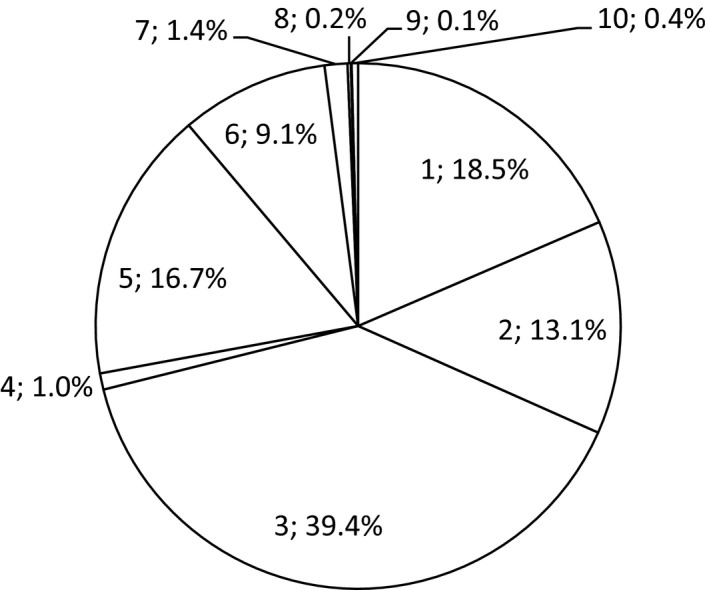
Proportions of planned nursing interventions (*N* = 2018). The figures represent: 1. dietary support, 2. pharmaceutical review, 3. nutritional treatment, 4. registration, 5. weight control, 6. oral care, 7. information/education regarding nutrition, 8. other intervention—malnutrition, 9. care in the final stages of life—malnutrition, 10. and declining all preventive interventions—malnutrition

**Figure 3:**
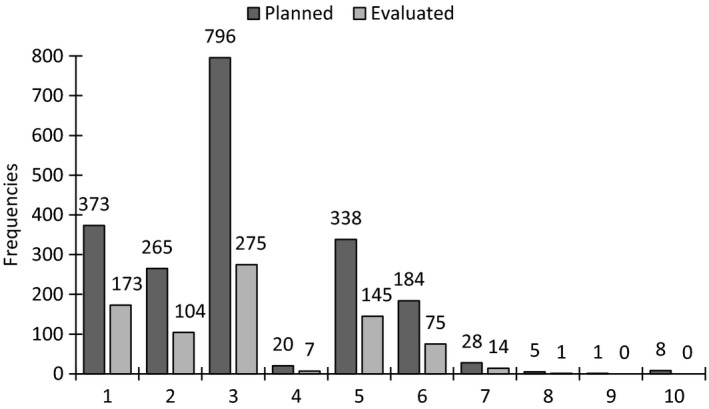
Frequencies of planned and evaluated nursing interventions. The figures on the x axis represent planned and evaluated interventions regarding: 1. dietary support, 2. pharmaceutical review, 3. Nutritional treatment, 4. registration, 5. weight control, 6. oral care, 7. information/education regarding nutrition, 8. other intervention—malnutrition, 9. care in the final stages of life—malnutrition, 10. and declining all preventive interventions—malnutrition

Among the 587 participants, 2,018 interventions were planned, the distribution of which is reported in percentages presented in Figure 2. The most common intervention was nutritional treatment i.e. snacks, enrichment of food, protein and energy enrichment, texture adaptation, nutritional supplements, adaptation of food according to culture or religion, overnight fasting decreased to a maximum of 11 hr, enteral nutrition and other intervention. The second most common intervention was dietary support i.e. adaptation of environment for individual meal situation, encouragement and prompting, feeding, planning aids and eating and drinking practice. The least common intervention was care in the final stages of life regarding malnutrition. Figure 3 presents frequencies of planned and evaluated nursing interventions and it shows that less than half of the interventions were evaluated in all the categories.

A logistic regression model with evaluated intervention as dependent variable adjusted for sex, age and BMI identified the following interventions as positive predictors of evaluated intervention: dietary support (*p* = .003 OR 2.16 CI 1.31), pharmaceutical review (*p* = .001, OR 2.08, CI 1.38–3.14) and weight control (*p* = .001, OR 3.94, CI 2.26–6.86), which indicates that these three interventions were more likely to be evaluated.

## 5 DISCUSSION

The current study showed that there were significant sex‐related differences in mean age and in BMI, but not in MNA‐SF score. However, a larger proportion of women were at risk of malnutrition. Those participants at risk of malnutrition did not have planned nursing interventions; further, in that risk group the men were more likely than the women to have planned interventions. The most common interventions were nutritional treatment, dietary support and weight control. Less than half of the planned nursing interventions were evaluated. Only one person had planned interventions for care in the final stage of life. It can be speculated if this reflects reality or if lack of time for documentation had an impact on the numbers of planned interventions and evaluations.

There were sex‐related differences in MNA‐SF scores i.e. a larger proportion of women being at risk of malnutrition, which is in line with previous research showing that malnutrition tends to be more common among women (Donini et al., 2013; Vandewoude & van Gossum, 2013). A master's thesis demonstrated similar sex‐related differences as in our study (Due‐Boje & Larsson, 2014). One explanation for this finding could be that women were significantly older than men and older age constitutes a risk factor for malnutrition.

The current study demonstrated that as less than half of the interventions were evaluated. One explanation for this finding may be that nurses due to heavy workload are forced to deprioritize documentation and evaluation of nursing interventions (Persenius et al., 2008). Another explanation may be reluctance to base decisions on evidence, but instead rely on knowledge based on experience or not using the registry Senior alert for continually feedback. If evaluations are not performed, it is difficult to discern what can be improved. Consequently, not performing evaluations of planned interventions may endanger patient safety as it remains unknown whether the interventions were effective or not; nor is it clear how one should proceed with nutritional treatment. The current study shows an urgent need to initiate quality improvement, focusing on evaluations of nursing interventions. One important aspect for the achievement of quality improvement is education (Cronenwett et al., 2009), which is in line with previous research recommending a greater need for education for nurses concerning nutritional assessment and evaluation (Lorefält et al., 2011; Norman et al., 2008).

The current study demonstrated that some interventions were more likely than others to be planned, with nutritional treatment being the most popular. This finding is in line with previous research showing that although it is difficult to find strategies to ensure that older persons gain weight, enrichment of food can slow chronic weight loss (Leslie et al., 2013). Additionally, a Cochrane review noted that a small weight gain could be achieved by using protein and energy supplementations. This approach may reduce mortality in malnourished persons and also decrease complications of malnutrition (Milne, Potter, Vivanti, & Avenell, 2009). For most participants, one intervention was planned, which is contrasting recommendations stating that it is an advantage for each patient to have multiple interventions planned (Beck, Damkjaer, & Sorbye, 2010). Thereby, our findings suggest that different nursing interventions in older people at risk for malnutrition could be combined at a greater extent to improve outcome.

Only 9.1% of the risk assessments in the present study concerned the planned intervention oral care. It has been shown that impaired oral health is associated with a significantly lower BMI. Moreover, it is known that poor oral health is common in institutionalized persons (Mojon, Budtz‐Jorgensen, & Rapin, 1999) and oral fungus infection is a significant risk factor for mortality (Holst et al., 2013). It seems therefore, appropriate to evaluate oral health in persons who are known to be at risk of malnutrition (Holst et al., 2013; Sumi, Ozawa, Miura, Michiwaki, & Umemura, 2010) and oral care alone can serve to maintain the nutritional status of older people (Sumi et al., 2010). However, although nurses are aware that oral care is important, there is a problem with non‐compliance, as indicated by a study where individuals did not always comply with a planned oral health intervention (Beck et al., 2010). This suggests that the implementation of clinical practice guidelines regarding oral health, which also includes educational efforts for nurses, may be needed to benefit persons living in municipal residential care homes as it entails quality improvement and a better quality of life.

The present results show a significant difference in BMI between men and women but Doumit, Nasser, and Hanna (2014) claim there are no differences between men and women according to BMI. Identifying those at risk of malnutrition can be problematic because measurements of BMI are not always performed. Indeed, various investigators have used the BMI scale in different ways: in some cases only the definition of malnutrition has been applied, whereas in other cases both malnutrition and the risk of malnutrition have been used (Johansson et al., 2009; Leslie, Lean, Woodward, Wallace, & Hankey, 2006). This makes it more difficult to compare results. Furthermore, there can be age‐related difficulties in correctly estimating body height, an issue that can be avoided by measuring knee height (Gavriilidou, Pihlsgard, & Elmstahl, 2015) or arm span instead (Villaverde‐Gutierrez, Sanchez‐Lopez, Ramirez‐Rodrigo, & Ocana‐Peinado, 2015). It can also be questioned whether measurement of BMI alone can reflect the risk of malnutrition (Riobo Servan et al., 2015). BMI is important as a single marker for the risk of malnutrition in people and is often routinely documented for residents of municipal care homes. However, such data can be misleading for individuals who have a high BMI and are losing weight, or for those who have had a low BMI their entire life (Stange et al., 2013). For instance, Winter, Flanagan, McNaughton, and Nowson (2013) identified risk for malnutrition among older people having a BMI indicating overweight or obesity. BMI can be questioned and speculated regarding its accuracy when it comes to older people and height estimation; this might have influenced the results in the current study.

The number of planned interventions differed among the evaluated persons in the current study and the majority of the older people had only one intervention. A single intervention is clearly not enough to improve the nutritional status of older people (Westergren & Hedin, 2010; Wikby, Ek, & Christensson, 2009) and it is likely that it will be necessary to use a combination of nursing interventions to achieve that goal. In the present study, there is no knowledge if more interventions were planned, but were not documented. When multiple interventions were planned, only less than half of the interventions were evaluated. It is essential that nurses at all levels realize the significance of documentation and evaluation; this might be achieved through educating them and through highlighting the importance of using the quality registries in quality improvements. Nurses' obligations not only include being in charge of the nursing process and providing safe care but also include suggesting and initiating quality improvements. It is vital for these professionals to be able to assess, plan and evaluate actions and interventions. For example, it is possible that an entry in a patient's chart can alert nurses that an evaluation is needed. Moreover, it is also recommended that nurses collaborate with other healthcare professionals and of course with the older person and his/her relatives when planning nutritional interventions.

### 5.1 Limitations

The strength of the current study is that it is based on a rather large sample size representing risk assessments carried out over an entire year in all municipal nursing homes in a mid‐sized town in southern Sweden. This may indicate that the current findings are representative for other nursing homes. A potential weakness may be that the risk assessment of malnutrition in Senior Alert is based on assessment through a questionnaire, which could increase the risk for under‐ or overrepresentation of risks for malnutrition (Edvinsson et al., 2015). However, MNA‐SF is a validated well‐known instrument developed from the MNA. Moreover, it is easy to use and highly reliable (Vellas et al., 2006) which could be regarded as a strong point in the current study. Data from quality registries like Senior Alert do not contain any medical or background data of the study participants, which could be regarded as a shortcoming because such data could be of importance for the interpretation of the results and could have provided a broader perspective on malnutrition in older people. However, a positive aspect of using data from Senior alert is that the result generates knowledge about the types of improvements that are needed to increase the quality of care for older people.

## 6 CONCLUSION

- This study revealed some significant differences between older men and women regarding BMI, risk of malnutrition and nursing interventions
- Importantly, the results showed that although interventions in older people at risk for malnutrition are often planned, they are not likely to be evaluated
- Not all persons at risk for malnutrition had planned interventions
- The current study argues that there is room for quality improvement concerning registration of interventions and evaluating interventions in older people at risk for malnutrition
- Hopefully, such quality improvement leads to interventions regarding malnutrition being performed in the clinical work, which in turn could increase the quality of care in older people living in municipal residential care homes and decrease the number of older people with malnutrition.

## ETHICAL APPROVAL

The study was approved by the Ethical Review Board of Malmö University (reference number: HS60‐2014/1244:5).

## PATIENT CONSENT

Swedish national quality registries such as Senior Alert are regulated by Swedish law—Patientdatalagen (2008) stipulating that personal data may not be processed in a quality registry such as Senior Alert, if the individual is opposed to it. The individual has the legal right to have personal information wiped out of the registry at any time (2008:355). Prior to registration, the healthcare provider has the legal obligation to inform the patient about the registry and data collection (2008:355; Edvinsson et al., 2015).

## CONFLICT OF INTEREST

No conflict of interest has been declared by the authors.

## AUTHOR CONTRIBUTIONS

AB and OH contributed equally to the study design, data collection and analyses and drafting the manuscript. CK contributed substantially to the study by drafting the manuscript, critical revision for important intellectual content and supervision. MA contributed substantially to the study conception and design, drafting the manuscript, critical revision for important intellectual content and supervision.
